# Antimicrobial peptides sourced from post-butter processing waste yak milk protein hydrolysates

**DOI:** 10.1186/s13568-017-0497-8

**Published:** 2017-12-06

**Authors:** Jinjin Pei, Hai Jiang, Xinsheng Li, Wengang Jin, Yanduo Tao

**Affiliations:** 1 Shaanxi Key Laboratory of Biology and Bioresources, College of Bioscience and Bioengineering, Shaanxi University of Technology, Chaoyang Rd, Hanzhong, 723001 Shaanxi China; 2 Key Laboratory of Tibetan Medicine Research, Northwest Institute of Plateau Biology, Chinese Academy of Sciences Xining, Xining, 810081 China; 3 Qinghai Key Laboratory of Tibetan Medicine Research, Northwest Institute of Plateau Biology, Chinese Academy of Sciences, Xining, 810081 China

**Keywords:** Antimicrobial peptides, Yak milk, Hydrolysates, Isolation

## Abstract

Yak butter is one of the most important foods for the Tibetan people. Of note, its production yields waste yak milk as a by-product. In this work, waste yak milk protein hydrolysates made via Pepsin hydrolysis were shown to have antimicrobial activity. Furthermore, an innovative method of magnetic liposome adsorption combined with reversed-phase high performance liquid chromatography (RP-HPLC) was developed to screen for and purify the antimicrobial peptides. Two antimicrobial peptides were obtained and their amino acid sequences were determined by N-sequencing, namely Arg-Val-Met-Phe-Lys-Trp-Ala and Lys-Val-Ile-Ser-Met-Ile. The antimicrobial activity spectra of Arg-Val-Met-Phe-Lys-Trp-Ala included *Bacillus subtilis*, *Staphylcoccus aureus, Listeria innocua, Escherichia coli, Enterobacter cloacae* and *Salmonella paratyphi*, while the Lys-Val-Ile-Ser-Met-Ile peptide shows not only bacterial growth inhibition but also of fungi. Haemolytic testing suggested that these two antimicrobial peptides could be considered to have no haemolytic effect at their minimum inhibitory concentrations (MICs).

## Introduction

Antimicrobial peptides are advantageous due to their non-toxic nature, high antimicrobial activity and good selectivity (Xiao and Zhang 2012; Li et al. 2013; Martinez et al. 2015). Thus, they hold promise for application as antimicrobial agents in the food and medical industries (Kaur et al. 2013; Zhang et al. 2016; Zhao et al. 2016). Yaks have been domesticated for 1000 of years and primarily kept for their milk, fibre, meat and blood. It has long been believed by locals that drinking yak milk can cure intestinal and stomach inflammation (Bartels et al. 1963; Singh and Sharma 2000). Moreover, the medicinal value of yak milk is believed to be derived from the many herbs (which themselves have medicinal properties) naturally occurring in the highlands that form part of the yaks’ natural diet (Bartels et al. 1963; Singh and Sharma 2000; Xin et al. 2016). Yak milk is mainly used to produce yak butter, which is the most important food for the Tibetans. However, the remaining yak “buttermilk” after making the butter is considered a waste by-product and often poured into the environment. Therefore, developing a technological application for waste yak milk (WYM) will have environmental benefits as well as a significant improvement to the local economy. Despite the isolation of antimicrobial peptides having been reported from many other sources (Kaur et al. 2013; Martinez et al. 2015; Zhao et al. 2016), there currently exists no reports about their isolation from yak milk. In this study, antimicrobial peptides were screened and purified from the WYM left after butter processing using a novel method of magnetic liposome adsorption combined with RP-HPLC. Furthermore, an additional primary objective of this study was the characterizations of the antimicrobial peptides.

## Materials and methods

### Waste yak milk hydrolysates preparation

The WYM left after butter processing was provided by a local yak butter processing factory. Following the procedure reported by Tang et al. (2014), the WYM was concentrated by rotary evaporation until the concentration was ten times greater. The concentrated WYM was then hydrolysed by the addition of Pepsin, Trypsin, Neutrase, Papain or Alcalase, as described by Tomasinsig et al. (2012). Each enzymatic hydrolysis was performed for 1, 2 and 3 h. The spot-on-lawn method was applied to determine the antimicrobial activity using *Staphylcoccus aureus* CICC 10384 as indicator stains (Yue et al. 2013).

### Nanomagnetic liposome preparation

Ferromagnetic Fe_3_O_4_ nanoparticles were prepared by the hydrothermal method (Duan et al. 2009). Egg-yolk phosphatidylcholine (EYPC, min 98% pure) and 1,2-dimyristoyl-sn-glycero-phosphatidylglycerol (sodium salt) (DMPG, min 97% pure) (2:1, w/w) were dissolved in chloroform/methanol (2:1, v/v) and nano-magnetic liposome particles were prepared by thin film dispersion (Paiva et al. 2012).

### Antimicrobial peptide screening and purification

Waste yak milk protein solutions were mixed and incubated with magnetic liposomes at 37 °C for 24 h so as to adsorb the active peptides out of solution. The magnetic liposomes were then isolated at 25 °C using a magnet column where PBS (pH 7.2, 10 mM) with 50 mM NaCl served as an isocratic mobile phase. Absorbance was recorded at 215 nm using a UV/Vis DAD (Agilent 1260 series, Agilent Technologies, Santa Clara, CA, USA). Peaks were collected and analysed for their potential bactericidal effect by the spot-on-lawn method with *S. aureus* CICC 10384 as indicator strains (Yue et al. 2013). The protein concentration was tested by Bradford analysis (Miao et al. 2016). The fractions with the highest antimicrobial activity were collected. Experiments were repeated several times to obtain a large amount of active elute, which was then concentrated by freeze drying (Tang et al. 2014). Further analysis and purification was conducted using RP-HPLC method (Waters Symmetry C18 column, 250 × 4.6 mm, 5 μm, Dublin, Ireland) with a gradient separation (mobile phase B: 5–100%) at a flow rate of 0.5 mL/min at 25 °C. Two mobile phases were used: mobile phase (A) 0.05% (v/v) TFA and mobile phase (B) 100% acetonitrile.

### Structural characterization

The purified antimicrobial peptides were sequenced by N-amino acid sequencing (Procise491, ABI, USA). Next, a BLAST analysis of their sequences was performed using the NCBI database (http://www.ncbi.nlm.nih.gov/). Physicochemical properties were predicted using a bioinformatics tool (ProtParam in Expasy ProtParam) (Chaparro and Da Silva Junior 2016). The Hyperchem 7.5 software was used to calculate and predict the lowest energy state 3D model structure (Tang et al. 2014).

### Synthesis of antimicrobial peptide

Antimicrobial peptides were synthesized by Sengong Bioengineering Ltd. Co. (Shanghai, China) (Yue et al. 2013). MALDI-TOF/MS and HPLC were used to confirm peptide purity and identity.

### Antimicrobial activity

Activity spectra (100 μg/ml) were determined with the selected indicator strains as shown in Table 1. Minimum inhibitory concentrations (MICs) were determined by testing the OD_600_ of bacteria suspensions treated with different dilutions of antimicrobial peptides (Yue et al. 2013).

**Table 1:** Activity of antimicrobial peptides

Microorganism	MIC			
	KVISMI		RVMFKWA	
Gram positive bacteria	μg/mL	μM	μg/mL	μM
*Bacillus subtilis* CICC 10034	32	23.2	32	23.2
*Staphylcoccus aureus* CICC10306	8	11.6	8	11.6
*Staphylococcus aureus* CICC 10384	8	11.6	8	11.6
*Listeria innocua* CICC 10417	4	5.8	8	11.6
Gram negative bacteria				
*Escherichia coli* CICC10293	8	11.6	16	23.2
*Pseudomonas aeruginosa* CICC 21636	16	23.2	32	46.4
*Serratia marcescens* ATCC 4112	32	46.4	32	46.4
*Enterobacter cloacae* CICC 21539	16	23.2	32	46.4
*Salmonella paratyphi* CICC 10437	32	46.4	32	46.4
Fungi				
*Aspergillus niger* CICC 2124	NA	NA	NA	NA
*Candida albicans* CICC 1965	32	46.4	NA	NA
*Saccharomyces cerevisiae* CICC 1002	32	46.4	NA	NA

CICC: China Center of Industrial Culture Collection, ATCC: American Type Culture Collection, NA: No inhibitory activity

### Haemolytic testing

Haemolytic testing was conducted with the mice red blood cells (Babl/c, SPF) (provided by Northwest A & F University, Shaanxi, China) with PBS (10 mM, pH 7.2) as negative control and 0.1% Triton X-100 as a positive control (Lin et al. 2013).

### Statistics

Data were analyzed by ANOVA using SPSS 16.0 software (Tang et al. 2014). The data are presented as the mean ± standard deviation. The statistical significance was defined as a *P* value of less than 0.05.

## Results

### Activity of Waste yak milk hydro lysates

**Fig. 1:**
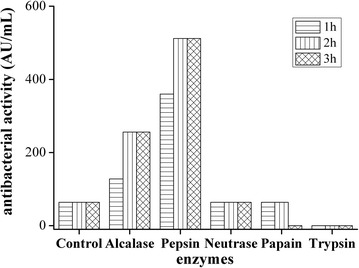
Activity of the hydrolysates sourced from waste yak milk

Fragments hydrolysed by Pepsin for 2 and 3 h exhibited the highest antimicrobial activities (Fig. 1). In the following experiments, yak blood fragments Pepsin hydrolysed for 2 h were used for subsequent antimicrobial peptide purification.

### Purification of antimicrobial peptides

**Fig. 2:**
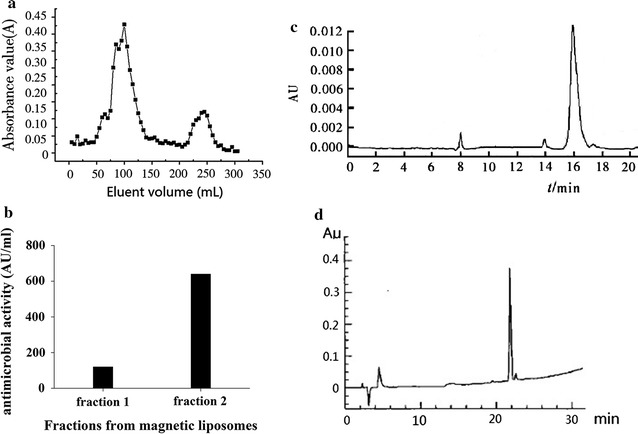
Purification of antimicrobial peptides from hydrolysates of waste yak milk. **a** Fractions from magnetic liposomes; **b** antimicrobial activity of fractions; **c** RP-HPLC spectrum of the purified antimicrobial peptide from elution #1; **d** RP-HPLC spectrum of the purified antimicrobial peptide from elution #2

Two fractions were eluted from the magnetic liposomes (Fig. 2a). Antimicrobial activity testing suggested that elution #2 showed the highest antimicrobial activity (Fig. 2b). These fractions were then further purified by RP-HPLC. As shown in Fig. 2c (Elution #1) and Fig. 2d (Elution #2), one signal peak can be observed, signifying a high purification of the antimicrobial peptide.

### Structural characterization of antimicrobial peptides

**Fig. 3:**
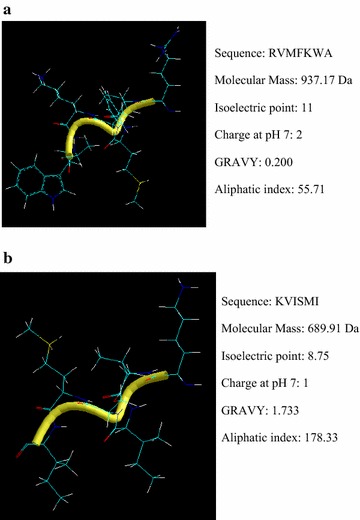
Structural characterization information and the theoretical structure of antimicrobial peptides. **a** “RVMFKWA”; **b** “KVISMI”

The amino acid sequences of these two antimicrobial peptides were successfully identified as Arg-Val-Met-Phe-Lys-Trp-Ala and Lys-Val-Ile-Ser-Met-Ile. However, no similarity with any known protein could be detected after performing BLAST analysis (http://web.expasy.org/blast/). Structural characterization and theoretical structures are predicted in Fig. 3. The theoretical minimum energy state for “RVMFKWA” is Energy = − 0.8208; Gradient = 0.0957, while that of “KVISMI” is Energy = 4.3906; Gradient = 0.0996.

### Antimicrobial activity of peptides

The activity spectra and MIC of these two antimicrobial peptides are listed in Table 1. “RVMFKWA” had growth inhibitory activity towards *Bacillus subtilis, Staphylcoccus aureus, Listeria innocua, Escherichia coli, Pseudomonas aeruginosa, Serratia marcescens, Enterobacter cloacae, Salmonella paratyphi* and *Shigella dysenteriae*. “KVISMI” was not only able to inhibit bacterial growth, but also the growth of some fungi, such as *Candida albicans* and *Saccharomyces cerevisiae*.

### Haemolytic activity

**Fig. 4:**
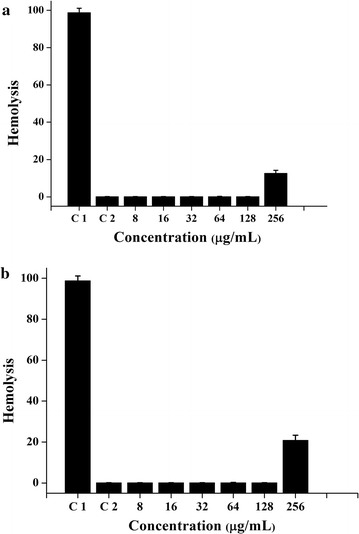
Hemolysis of antimicrobial peptides. **a** “RVMFKWA”; **b** “KVISMI”. C1: control 1, Triton X-100 instead of the antimicrobial peptide; C2: control, PSB instead of the antimicrobial peptide

The haemolytic activities of these two antimicrobial peptides are shown in Fig. 4. Nearly undetectable haemolysis is observed at the MIC. When concentrations more than 256 μg/ml were used, 12.63 and 20.81% haemolysis was observed using “RVMFKWA” and “KVISMI” respectively. These data demonstrate that these two antimicrobial peptides have no haemolytic activity near their MICs.

## Discussion

Many studies have investigated the isolation of high activity antimicrobial peptides from hydrolysates obtained via different enzyme treatments (Yang et al. 2012; Kobbi et al. 2015; Sun et al. 2016). For example, Bah reported the use of proteases to hydrolyse deer, sheep, pig and cattle red blood cell fractions to successfully obtain antimicrobial peptides (Bah et al. 2016).

Traditional peptide purification procedures have many disadvantages; they are lengthy and complex to perform, exhibit a low recovery of desired products and a loss of activity (Kandula and Terli 2013; Goh and Philip 2015; Panagiota et al. 2016; Wang et al. 2016). In this study, the adsorption onto magnetic liposomes combined with RP-HPLC was able to quickly screen and isolate potential antimicrobial components.

Mammals are one of the most important sources of antimicrobial peptides (Expósito et al. 2006; Stanford et al. 2009). Expósito et al. (2006), identified bovine antibacterial peptides. Bu et al. (2011), reported the discovery of an antimicrobial peptide from Kulun donkey blood. However, until now there has been no study regarding the isolation of antimicrobial peptides from WYM. These two antimicrobial peptides both have a low molecular mass (937.17 and 689.91 Da). Earlier studies implied that antimicrobial peptides are generally low-molecular-weight, such as SP-1 (MW 878.97 Da) (Sun et al. 2016), and Glu-Leu-Ala–Ala-Ala-Cys (MW 162.1 Da) (Kobbi et al. 2015), showed strong antimicrobial activity.

Ma et al. (2016), reported that antimicrobial peptides isolated from whey were able to inhibit the growth of *Streptococcus agalactiae, Staphylococcus aureus,* and *Escherichia coli*, but not fungi. “KVISMI” demonstrated antifungi activity that was not seen in Ma et al. (2016). Compared with the antimicrobial activity of the bovine-derived peptides reported by Expósito et al. (2006), “KVISMI” showed higher potency against *Staphylococcus aureus* and *Escherichia coli*.

Typically, antimicrobial peptides exhibiting no/low haemolytic activity can be considered for practical application (Wang et al. 2016). For example, Ma reported that their antimicrobial peptide derived from swine blood was able to induce 2.4% haemolysis at the MICs (Ma et al. 2016). The results in this study indicated that the two antimicrobial peptides could be considered to have no haemolytic at MICs.

## Abbreviations

RP-HPLC: reversed-phase high performance liquid chromatography
K: Lys
Y: Tyr
G: Gly
N: Asn
L: Leu
S: Ser
R: Arg
I: Ile
F: Phe
A: Ala
V: Val
M: Met
W: Trp
